# Molecular detection of novel *Anaplasma *sp *. *and zoonotic hemopathogens in livestock and their hematophagous biting keds (genus *Hippobosca*) from Laisamis, northern Kenya

**DOI:** 10.12688/openresafrica.13404.1

**Published:** 2022-06-06

**Authors:** Daniel M. Mwaki, Kevin O. Kidambasi, Johnson Kinyua, Kenneth Ogila, Collins Kigen, Dennis Getange, Jandouwe Villinger, Daniel K. Masiga, Mark Carrington, Joel L. Bargul

**Affiliations:** 1 Animal Health Department/Molecular Biology and Bioinformatics Unit, International Centre of Insect Physiology and Ecology (icipe), Nairobi, P.O. BOX 30772-00100, Kenya; 2 Department of Biochemistry, Jomo Kenyatta University of Agriculture and Technology (JKUAT), Nairobi, P.O. BOX 62000-00200, Kenya; 3 Department of Zoology, Jomo Kenyatta University of Agriculture and Technology (JKUAT), Nairobi, P.O. Box 62000-00200, Kenya; 4 Department of Biochemistry, University of Cambridge, Tennis Court Road, Cambridge, CB2 1QW, UK

**Keywords:** Vector-borne diseases, Hippobosca, high-resolution melting analysis, hemopathogens, keds

## Abstract

**Background:** Livestock are key sources of livelihood among pastoral communities. Livestock productivity is chiefly constrained by pests and diseases. Due to inadequate disease surveillance in northern Kenya, little is known about pathogens circulating within livestock and the role of livestock-associated biting keds (genus *Hippobosca*) in disease transmission. We aimed to identify the prevalence of selected hemopathogens in livestock and their associated blood-feeding keds.

**Methods:** We randomly collected 389 blood samples from goats (245), sheep (108), and donkeys (36), as well as 235 keds from both goats and sheep (116), donkeys (11), and dogs (108) in Laisamis, Marsabit County, northern Kenya. We screened all samples for selected hemopathogens by high-resolution melting (HRM) analysis and sequencing of PCR products amplified using primers specific to the genera: *Anaplasma, Trypanosoma, Clostridium, Ehrlichia, Brucella, Theileria,* and *Babesia.*

**Results:** In goats, we detected *Anaplasma ovis* (84.5%), a novel *Anaplasma *sp. (11.8%), *Trypanosoma vivax* (7.3%), *Ehrlichia canis* (66.1%), and *Theileria ovis* (0.8%). We also detected *A. ovis *(93.5%), *E. canis *(22.2%), and *T. ovis *(38.9%) in sheep. In donkeys, we detected ‘ *Candidatus *Anaplasma camelii’ (11.1%), *T. vivax* (22.2%), *E. canis* (25%), and *Theileria equi *(13.9%). In addition, keds carried the following pathogens; goat/sheep keds - *T. vivax* (29.3%) *, Trypanosoma evansi* (0.86%), *Trypanosoma godfreyi *(0.86%), and *E. canis *(51.7%); donkey keds - *T. vivax* (18.2%) and *E. canis *(63.6%); and dog keds - *T. vivax *(15.7%), *T. evansi* (0.9%), *Trypanosoma simiae *(0.9%) *,* *E. canis *(76%), *Clostridium perfringens *(46.3%), *Bartonella* *schoenbuchensis *(76%), and *Brucella abortus* (5.6%).

**Conclusions:** We found that livestock and their associated ectoparasitic biting keds carry a number of infectious hemopathogens, including the zoonotic *B. abortus*. Dog keds harbored the most pathogens, suggesting dogs, which closely interact with livestock and humans, as key reservoirs of diseases in Laisamis. These findings can guide policy makers in disease control.

## Introduction

Livestock in Africa are considered as one of the most valuable agricultural assets for the rural and urban poor, and accounts for about 40% of the agricultural GDP ( Malabo Montpellier Panel, 2020). In 2018, Africa’s total livestock population was estimated at 2 billion poultry birds, 438 million goats, 384 million sheep, about 356 million cattle, 40.5 million pigs, almost 31 million camels, and 38 million equines (including 30 million donkeys, 6.5 million horses, and 885,000 mules) ( Malabo Montpellier Panel, 2020). This represents about one-third of the world’s livestock population ( Otte *et al.*, 2019). Moreover, livestock production plays a key economic role to the livelihood of pastoralists living in the marginalized arid and semi-arid regions of northern Kenya ( Mburu *et al.*, 2017). These pastoralists largely depend on livestock as a source of meat and milk, income from selling livestock, and donkeys and camels also serve as a mode of transport. Pastoralism supports about 20 million people, produces about 90% of the meat consumed in East Africa and contributes to about 13% of the GDP in Kenya ( Nyariki & Amwata, 2019).

Unfortunately, livestock production is hindered by pests and diseases, which are endemic in northern Kenya ( Perry & Grace, 2009). Hemopathogens of livestock, particularly those of zoonotic importance, are responsible for some of the most serious emerging infectious diseases facing sub-Saharan Africa and the rest of the world ( Rosenberg *et al.*, 2018). About 75% of newly emerging diseases currently affecting humans originated in animals ( Jones *et al.*, 2008). In Kenya, hemoparasites that cause babesiosis, theileriosis, rickettsiosis, anaplasmosis, and ehrlichiosis are a major impediment to livestock productivity and public health ( Kiara *et al.*, 2014).

Bacterial diseases in livestock include bartonellosis caused by *Bartonella* spp. *,* which is mainly transmitted by biting arthropod vectors such as ticks and reported widely in both wild and domestic mammals such as dogs, cats, and cattle ( Ereqat *et al.*, 2016). In addition, brucellosis, a zoonotic disease, has been reported worldwide and mainly causes infections to the genitals of animals, abortion, and fetal death ( Probert *et al.*, 2004). *Brucella* species have been shown to be of high public health and socio-economic importance in northern Kenya ( Kairu-Wanyoike *et al.*, 2019). Parasitic protozoal infections, for example African animal trypanosomiasis, cause debilitating diseases in livestock and serious economic losses in Africa ( Petersen *et al.*, 2007). Etiological agents such as *Clostridium perfringens* cause enteric diseases such as enterotoxaemia in both humans and livestock, mostly goats and sheep ( Singh *et al.*, 2018).

Livestock act as reservoirs of infectious pathogens that can be transmitted by various vectors. Ticks and biting flies such as *Stomoxys* spp. and tabanids are vectors of infectious pathogens including bacteria, viruses ( *e.g.*, Rift Valley fever viruses), rickettsiae ( *Coxiella, Anaplasma*), and protozoa ( *T. evansi, T. vivax*, *T. simiae*) ( Baldacchino *et al.*, 2013; Narladkar, 2018). Hippoboscid flies, commonly known as keds and belonging to the family Hippoboscidae within the superfamily Hippoboscoidea, are obligate ectoparasites of vertebrates, both domestic and wild animals and birds ( Petersen *et al.*, 2007; Rahola *et al.*, 2011). Members of Hippoboscidae act as vectors of many infectious agents including bacteria, viruses, and protozoans ( Rahola *et al.*, 2011). Keds cause economic losses in various ways, including annoyance and psychological disturbances produced during the act of biting and feeding, the diseases they transmit ( Bargul *et al.*, 2021), and expenditure incurred by farmers in controlling them ( Narladkar, 2018). The painful bites inflicted on the bloodmeal host by keds result in skin lesions and by feeding on blood, they contribute to anaemia ( Oyieke & Reid, 2003).

In northern Kenya, keds and ticks are common external pests of livestock, found on livestock all year round ( Bargul *et al.*, 2021). Keds are known to infest and blood-feed on all livestock species, domestic and wild animals. In addition, keds also feed on humans and in the process, could transmit zoonotic pathogens ( Getahun *et al.*, 2020). To date, little efforts have been put into surveillance of pathogens harbored by the livestock and the role of keds in spreading various diseases. This calls for an urgent need for research studies to catalogue livestock infectious and zoonotic pathogens circulating in livestock for a better understanding of disease prevalence, transmission routes, and for control. In this study, we screened for selected hemopathogens ( *Anaplasma, Trypanosoma, Clostridium, Ehrlichia, Brucella, Theileria,* and *Babesia* spp.) in goats, sheep, and donkeys, and in keds collected from goats, sheep, dogs, and donkeys.

## Methods

### Study site

**Figure 1.**
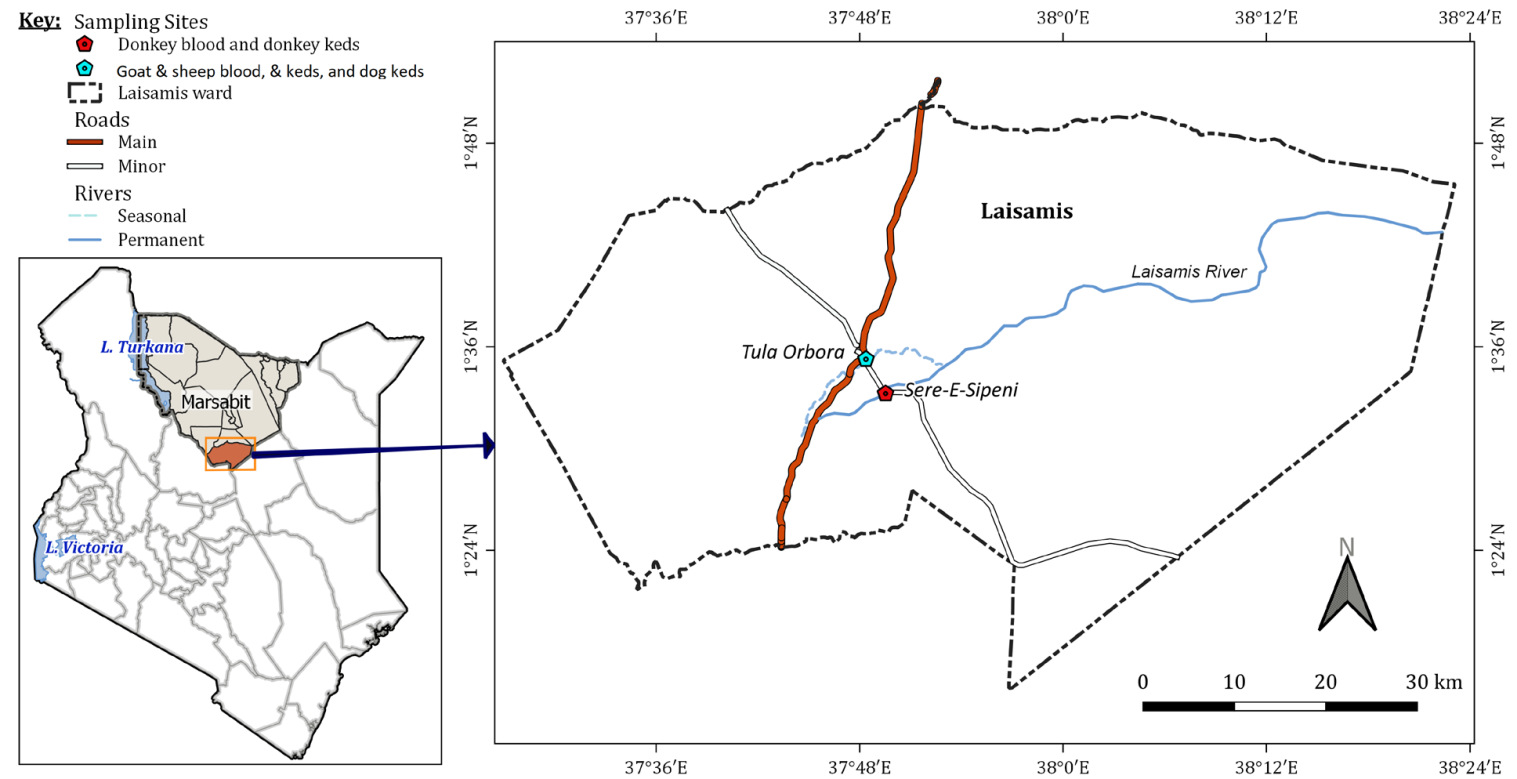
A map of Kenya showing the study sites in Laisamis, Marsabit County. The samples collected from each site are shown on the map key.

The study was conducted in Laisamis sub-County (1° 36' 0" N, 37° 48' 0" E) in Marsabit County, northern Kenya ( Figure 1). Marsabit County borders Ethiopia to the North, Turkana County to the West, Samburu and Isiolo Counties to the South, and Wajir County to the East. Laisamis sub-County occupies an area of 20,290 km ^2^ that comprises five County Assembly Wards, among which Laisamis Ward (3,885 km ^2^), the area of this study has arid and semi-arid climatic conditions ( Marsabit CIDP, 2018). The main economic activity in this region is livestock rearing with limited crop production. The main livestock species kept in Marsabit County include approximately 217,360 camels, 2,029,490 goats, 1,851,452 sheep, 420,000 cattle, 81,900 donkeys, and 45,860 poultry ( Marsabit CIDP, 2018).

### Sample collection

Samples were collected in two field-sampling trips and each sampling site was geo-referenced with a global positioning system (GPS). Goat and sheep blood, keds on goats, sheep, and dog keds were collected in July 2019 along the Laisamis River at Tula Orbora, which is one of the main livestock watering points (1° 35’ 16.4” N, 37° 48’ 22.5” E). Donkey blood and donkey keds were collected at Sere-e-Sipeni (1°33’14.9” N, 37°49’32.1” E) in February 2020. 

### Ethical approval

This study was conducted in strict adherence to the experimental guidelines and procedures approved by the International Centre of Insect Physiology and Ecology (icipe *)* Institutional Animal Care and Use Committee (REF: IACUC/ICIPE/003/2018) and the Pwani University Ethics Review (approval number: ERC/EXT/002/2020). Goats, sheep, and donkeys were handled carefully to minimize pain and discomfort. Verbal consent was obtained from livestock owners prior to collection of samples. Written consent was not possible as the livestock keepers could neither read nor write.

### Blood collection

About 5 mL of blood was obtained from the jugular vein of 245 goats (22 males and 223 females), 108 sheep (8 males and 100 females), and 36 donkeys (18 males and 18 females) of both sexes. Each sample was collected into 5 mL EDTA vacutainers (Plymouth PLG, UK), and kept under cold chain during the sampling exercise. Immediately after completion of the sampling process, all blood samples were preserved in liquid nitrogen for transportation to *icipe*, Nairobi, for molecular detection of pathogens. 

### Collection and identification of livestock keds

Keds that infested goats, sheep, donkeys, and dogs were collected from their hosts by handpicking at night as previously reported ( Kidambasi *et al.*, 2020). Freshly collected keds were preserved in absolute ethanol ready for transportation to *icipe* for molecular screening of pathogens. Keds for use in molecular and morphological identification were sorted at *icipe* (Nairobi). Species identification based on morphology was done through comparison with known hippoboscid collections at *icipe*.

### DNA extraction

Keds were surface-sterilized with 70% ethanol and left to air dry for 10 min on a paper towel in a clean hood. Each fly was then placed into a clean 1.5-mL Eppendorf tube containing 250 mg of sterile zirconia beads of 2-mm diameter (Stratech, UK). The flies were homogenized in liquid nitrogen using a Mini-Beadbeater-16 for 3 min (BioSpec, Bartlesville, OK, USA). Genomic DNA was extracted from individual flies and blood samples using DNeasy Blood & Tissue Kit (Qiagen, Hilden, Germany), following the manufacturer’s instructions.

### PCR-HRM for pathogen detection

Hemopathogens including *Anaplasma*, *Ehrlichia*, piroplasms ( *Theileria* and *Babesia* spp.), animal African trypanosomes, *Clostridium perfringens* and *Brucella* spp. were amplified using pathogen-specific PCRs ( Table 1) followed by DNA fragment analysis based on high-resolution melting (HRM) analysis. Rotor-Gene Q (Qiagen, Hannover, Germany), Quant Studio 3 Real-Time PCR System (Applied Biosystems, Foster City, CA, USA) and Mic qPCR (Bio Molecular Systems, Upper Coomera, Queensland, Australia) thermocyclers were used for PCR-HRM analysis for pathogen detection.

**Table 1.** PCR primers for pathogen detection.

Primer name	5’ to 3’ sequence	Target organism	Target gene	Amplicon size (bp)	Primer reference
*Anaplasma*JV_F *Anaplasma*JV_R	CGGTGGAGCATGTGGTTTAATTC CGRCGTTGCAACCTATTGTAGTC	*Anaplasma* spp.	Partial 16S rRNA	300	( Mwamuye *et al.*, 2017)
*Ehrlichia*JV_F *Ehrlichia*JV_R	GCAACCCTCATCCTTAGTTACCA TGTTACGACTTCACCCTAGTCAC	*Ehrlichia* spp.	16S rRNA	300	( Mwamuye *et al.*, 2017)
*Ehrlichia 16S* F *Ehrlichia 16S* R	CGTAAAGGGCACGTAGGTGGACTA CACCTCAGTGTCAGTATCGAACCA	*Ehrlichia* spp.	16S rRNA	200	( Tokarz *et al.*, 2009)
EHR 16SD 1492R	GGTACCYACAGAAGAAGTCC GGTTACCTTGTTACGACTT	*Ehrlichia* and *Anaplasma* spp.	Longer 16S rRNA	1000	( Parola *et al.*, 2000)
ITS1_CF ITS1_BR	CCGGAAGTTCACCGATATTG TTGCTGCGTTCTTCAAC- GAA	*Trypanosoma* spp.	ITS1	250–720	( Njiru *et al.*, 2005)
RLB_F RLB_R	GAGGTAGTGACAAGAAATAACAATA TCTTCGATCCCCTAACTTTC	*Theileria* and *Babesia* spp.	18S rRNA	450	( Gubbels *et al.*, 1999)
Br_F Br_R	GCTCGGTTGCCAATATCAATGC GGGTAAAGCGTCGCCAGAAG	*Brucella* spp.	bcsp31	223	( Probert *et al.*, 2004)
Cp_F Cp_R	AAAGATGGCATCATCATTCAAC TACCGTCATTATCTTCCCCAAA	*Clostridium* *perfringens*	16S rRNA	279	( Wu *et al.*, 2009)

Genus-specific *Anaplasmataceae* primers were used for amplification of the 16S rRNA gene of *Ehrlichia* and *Anaplasma* spp. ( Mwamuye *et al.*, 2017), while *Theileria* and *Babesia* spp. were screened simultaneously using primers that target the hypervariable V4 region of the 18S rRNA gene ( Gubbels *et al.*, 1999). *Clostridium perfringens* was detected using specific primers targeting the 16S rRNA gene ( Wu *et al.*, 2009). A set of genus-specific primers described by ( Probert *et al.*, 2004) that targets the bcsp31 gene was used for identification of *Brucella* spp. A universal set of primer that targets the trypanosomal internal transcribed spacer I (ITS-I) region was used for detection of animal African trypanosomes ( Njiru *et al.*, 2005).

PCR-HRM assays were performed in runs of 10 μL reaction volumes, containing 6 μL nuclease-free water, 2 μL of 5×HOT FIREPol EvaGreen HRM mix (no ROX) (Solis BioDyne, Estonia), 0.5 μL of 10 pmol of each primer and 1 μL of template DNA.

PCR conditions in the Rotor-Gene, Quant Studio and Mic qPCR for detection of *Ehrlichia, Anaplasma* and piroplasms ( *Theileria* and *Babesia*) were preceded by an initial enzyme activation step at 95°C for 15 min followed by 10 cycles of denaturation at 94°C for 20 sec, touch-down annealing from 64°C with a decrease of 1°C after each cycle for 25 sec, and primer extension step at 72°C for 30 sec. Then, another 30 cycles each of: denaturation at 94°C for 20 sec, touch-down annealing from 55°C with a decrease of 1°C after every 5 cycles for 50 sec, and extension at 72°C for 30 sec, with a final elongation at 72°C for 3 min.

Specific annealing temperatures of 55°C, 53.9°C, and 63.2°C were used for detection of *Trypanosome* spp *., Clostridium perfringens*, and *Brucella* spp *.,* respectively. The PCR conditions were initial enzyme activation at 95°C for 15 min, 40 cycles of: denaturation at 95°C for 30 sec, annealing for 30 sec, and extension at 72°C for 30 sec, with a final elongation at 72°C for 7 min.

HRM analysis proceeded immediately after PCR with a gradual increase in temperature from 75°C to 95°C with 2 sec increase of 0.1°C between successive fluorescence acquisitions. The melting curves were visualized based on the fluorescence signals and the change in fluorescence with time (dF/dT) plotted against change in temperature (°C).

Rotor-Gene Q Series Software 2.1.0 (Build 9), Quant Studio Design and Analysis Software version 1.5.1 ( Mwamuye *et al.*, 2017), and micPCR Software v2.8.1 were used to assess melt profiles of the test samples in comparison with that of the known positive controls to confirm detection of pathogens. DNA sequencing of representative samples showing distinct melting curves proceeded to identify the pathogens.

### Purification of PCR amplicons and gene sequencing

Representative samples with expected and distinct melting curves relative to the known positive controls were amplified in larger PCR reaction volumes of 15 μL. Five μL of the PCR amplicons were resolved through 2% ethidium bromide-stained agarose gel electrophoresis followed by visualization of the DNA under ultraviolet light using Kodak Gel Logic 200 Imaging System (SPW Industrial, Laguna Hills, CA, USA). About 10 μL of each sample with clear bands was purified using ExoSAP-IT PCR Product Cleanup kit (Affymetrix, Santa Clara, CA, USA) following the manufacturer’s protocol. Purified samples were then incubated at 37°C for 15 min and 85°C for 15 min in a Proflex thermocycler (Applied Biosystems) prior to Sanger sequencing by Macrogen, Inc. (Amsterdam, Netherlands). All sequences generated by this study were deposited in the GenBank (NCBI) database and assigned accession numbers.

### Data analysis

The Rotor-Gene Q Series 2.1.0 (Build 9), Quant Studio Design and Analysis Software v1.5.1, and micPCR Software v2.8.1 were used for HRM analysis. Data on pathogens from blood and ked samples were recorded in a Microsoft Excel Spreadsheet Program version 18.2110.13110.0 (Microsoft Corp.).

All nucleotide sequences were edited and aligned with closely related sequences from the NCBI GenBank nr database using the MAFFT plugin in Geneious Prime software version 2020.2.1 (created by Biomatters, Auckland, New Zealand; open source alternatives: UGENE, BioEdit) ( Kearse *et al.*, 2012). The Basic Local Alignment Search Tool ( BLAST) was used to query related sequences available in the GenBank nr database. Maps showing the sampling sites were generated by feeding the coordinates of the sampling locations into a GIS software, QGIS v3.16.

### Phylogenetic analysis

Maximum likelihood phylogenetic trees of the sequence alignments were constructed using PhyML v3.0 ( Guindon *et al.*, 2010). Tree topologies were estimated using nearest neighbor interchange improvements over 1,000 bootstrap replicates and the Akaike information criterion for automatic model selection was employed in the phylogenies. FigTree v1.4.3 ( Rambaut, 2016) was used to visualize the phylogenetic trees.

## Results

### Detection of pathogens

A total of 389 blood samples from goats (245), sheep (108) and donkeys (36), as well as 235 keds from goats and sheep (116), dogs (108) and donkeys (11) were randomly collected in Laisamis Sub-County, Marsabit County of northern Kenya. Out of the 389 blood samples screened, 87.7% (341/389) tested positive for *Anaplasma* spp., 50.1% (195/389) tested positive for *Ehrlichia canis*, 12.6% (49/389) tested positive for *Theileria* spp., and 6.7% (26/389) tested positive for *Trypanosoma* spp.

Out of 235 ked samples screened, 63.4% (149/235) were positive for *E. canis*, 34.9% (82/235) were positive for *Bartonella schoenbuchensis,* 24.3% (57/235) were positive for *Trypanosoma* spp., 21.3% (50/235) were positive for *Clostridium perfringens*, and only 2.6% (6/235) were positive for *B. abortus.* All the pathogens detected in this study are listed in Table 2.

**Table 2.** Summary of hemopathogens detected in livestock and their keds.

Pathogens	Prevalence of hemopathogens in blood and ked samples					
	Goat blood (n=245)	Sheep blood (n=108)	Goat/sheep keds (n=116)	Dog keds (n=108)	Donkey blood (n=36)	Donkey keds (n=11)
*Trypanosoma* spp *.*	*Trypanosoma vivax* = 18 (7.3%)	__	*Trypanosoma * *vivax* = 34 (29.3%) *Trypanosoma * *evansi* = 1 (0.86%) *Trypanosoma * *godfreyi* = 1 (0.86%)	*Trypanosoma * *vivax* = 17 (15.7%) *Trypanosoma * *simiae* = 1 (0.9%) *Trypanosoma * *evansi* = 1 (0.9%)	*Trypanosoma * *vivax* = 8 (22.2%)	*Trypanosoma * *vivax* = 2 (18.2%)
*Anaplasma* spp.	*Anaplasma ovis* = 207 (84.5%) Novel *Anaplasma* sp. = 29 (11.8%)	*Anaplasma ovis* = 101 (93.5%)	__	__	‘ *Candidatus* *Anaplasma camelii’* = 4 (11.1%)	__
*Ehrlichia canis*	162 (66.1%)	24 (22.2%)	60 (51.7%)	82 (76%)	9 (25%)	7 (63.6%)
*Theileria/* *Babesia* spp.	*Theileria ovis* = 2 (0.8%)	*Theileria ovis* = 42 (38.9%)	__	__	*Theileria equi* = 5 (13.9%)	__
*Brucella abortus	__	__	__	6 (5.6%)	__	__
*Clostridium *perfringens*	__	__	__	50 (46.3%)	__	__
*Bartonella *schoenbuchensis*	__	__	__	82 (76%)	__	__

*Zoonotic pathogens; dash ( ^__^) means the pathogen was not detected.

### Pathogen detection in blood samples

**Figure 2.**
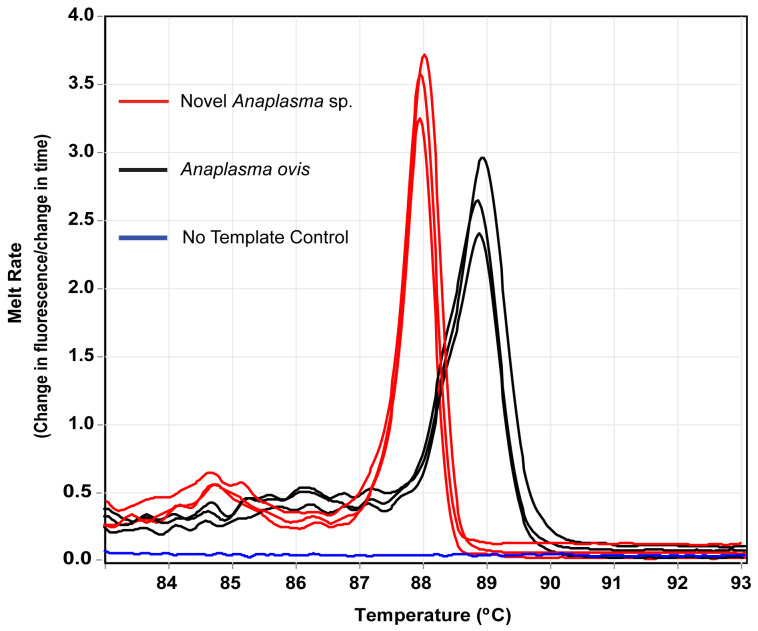
Melt curves of amplification of 16S rRNA of *Anaplasma* spp. in goats.

***Goat and sheep blood samples.*** In goat blood (245), we detected *Anaplasma ovis* 84.5% (207), novel *Anaplasma* sp. 11.8% (29), *E. canis* 66.1% (162), *Trypanosoma vivax* 7.3% (18), and *Theileria ovis* 0.8% (2) by PCR-HRM ( Figure 2).

We also detected *A. ovis* 93.5% (101), *E. canis* 22.2% (24), and *T. ovis* 38.9% (42) in sheep blood (108).

**Figure 3.**
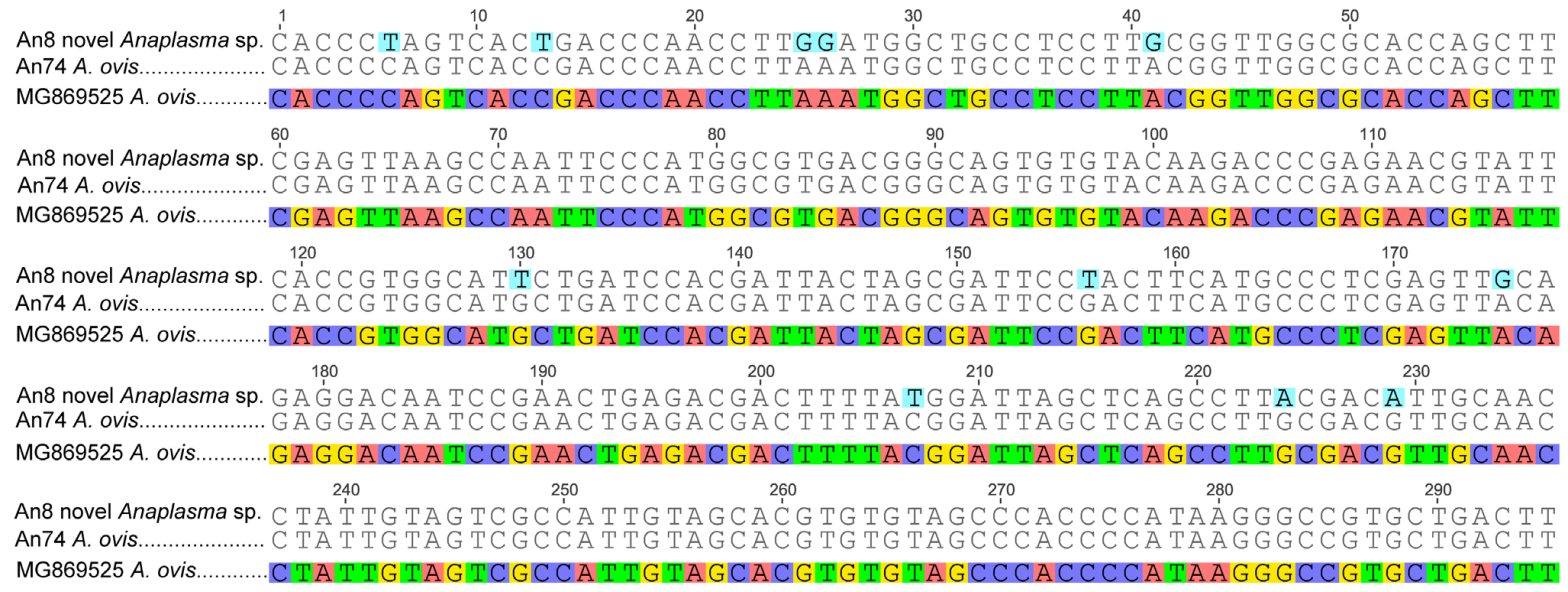
Multiple sequence alignment of 16S rRNA sequences of (i) novel *Anaplasma* sp. (from study sample An8) and (ii) *A. ovis* (An74) amplified from goat blood, and (iii) the GenBank-retrieved sequence of *A. ovis,* accession MG869525. Nucleotide changes were identified between MG869525 and An8 sequences. For instance, at position 25 and 26 in the above alignment, ‘AA’ in the GenBank sequence MG869525 is replaced by ‘GG’ in the query sequence An8 (study sample). In contrast, An74 sequence (from this study) was 100% identical to the *A. ovis* sequence MG869525 from GenBank.

Alignment of the edited *Anaplasma* 16S rRNA sequences with closely related sequences queried on NCBI GenBank nr database, showed that most samples were 100% identical to *A. ovis* (GenBank accession MG869525). However, some sequences were distinctly different from the queried *A. ovis* among other sequences with an identity of 96.8% and below ( Figure 3). In addition, alignment of the edited *Theileria* 18S rRNA sequences with closely related sequences showed 100% similarity to *T. ovis* (GenBank accession MN712508).

***Donkey blood samples.*** In donkey blood samples (36), we detected *E. canis* 25% (9), *‘Candidatus* Anaplasma camelii’ 11.1% (4), *Trypanosoma vivax* 22.2% (8) and *Theileria equi* 13.9% (5). Analysis of the 200-bp *Ehrlichia* 16S rRNA sequences showed 100% identity to *E. canis* sequenced from ticks and fleas collected from companion dogs and cats in East and Southeast Asia (GenBank accession MT499360). On the other hand, alignment of the edited *Theileria* 18S rRNA sequences with closely related sequences queried on NCBI GenBank nr database, showed 100% identity with *Theileria equi* (GenBank accession MK063829). Additionally, analysis of the *Anaplasma* 16S rRNA sequences showed 100% identity to *‘Candidatus* Anaplasma camelii’ (GenBank accession MT510533). *Brucella* spp. and *Clostridium perfringens* were not detected in donkey and goat blood.

### Pathogen detection in keds

***Goat and sheep keds.*** The keds collected from co-herded goats and sheep were found to harbor *E. canis* 51.7% (60/116) and three trypanosome species, namely *T. vivax* 29.3% (34/116), *Trypanosoma evansi* 0.86% (1/116) and *Trypanosoma godfreyi* 0.86% (1/116).

***Donkey keds.*** The pathogens that were detected in keds collected from donkeys included; *E. canis* 63.6% (7/11) and *T. vivax* 18.2% (2/11).

***Dog keds.*** We detected *E. canis* 76% (82/108), *T. vivax* 15.7% (17/108), *Trypanosoma simiae* 0.9% (1/108), and *T. evansi* 0.9% (1/108) in dog keds. Also, the dog keds harbored *Clostridium perfringens* 46.3% (50/108), *B. abortus* 5.6% (6/108) and *Bartonella schoenbuchensis* 76% (82/108).

Similarly, analysis of the 200-bp *Ehrlichia* 16S rRNA sequences showed 100% identity with *E. canis* sequenced from ticks and fleas collected from companion dogs and cats in East and Southeast Asia (GenBank accession MT499360). All the ked samples were negative for *Anaplasma* spp. and piroplasms ( *Theileria* and *Babesia* spp.). Moreover, only keds obtained from dogs were positive for *B. abortus*, *C. perfringens* and *B. schoenbuchensis.* The *Brucella* bcsp31 gene sequences showed a 98.68% identity with *B. abortus* sequenced from cattle milk DNA in India (GenBank accession MK881176).

The morphological identification of the keds matched with the molecular identification of two ked species: *Hippobosca variegata* (GenBank accession MW128366) and *Hippobosca longipennis* (GenBank accession MW128365).

### Phylogenetic analysis of Anaplasma spp. 16S rRNA sequences

**Figure 4.**
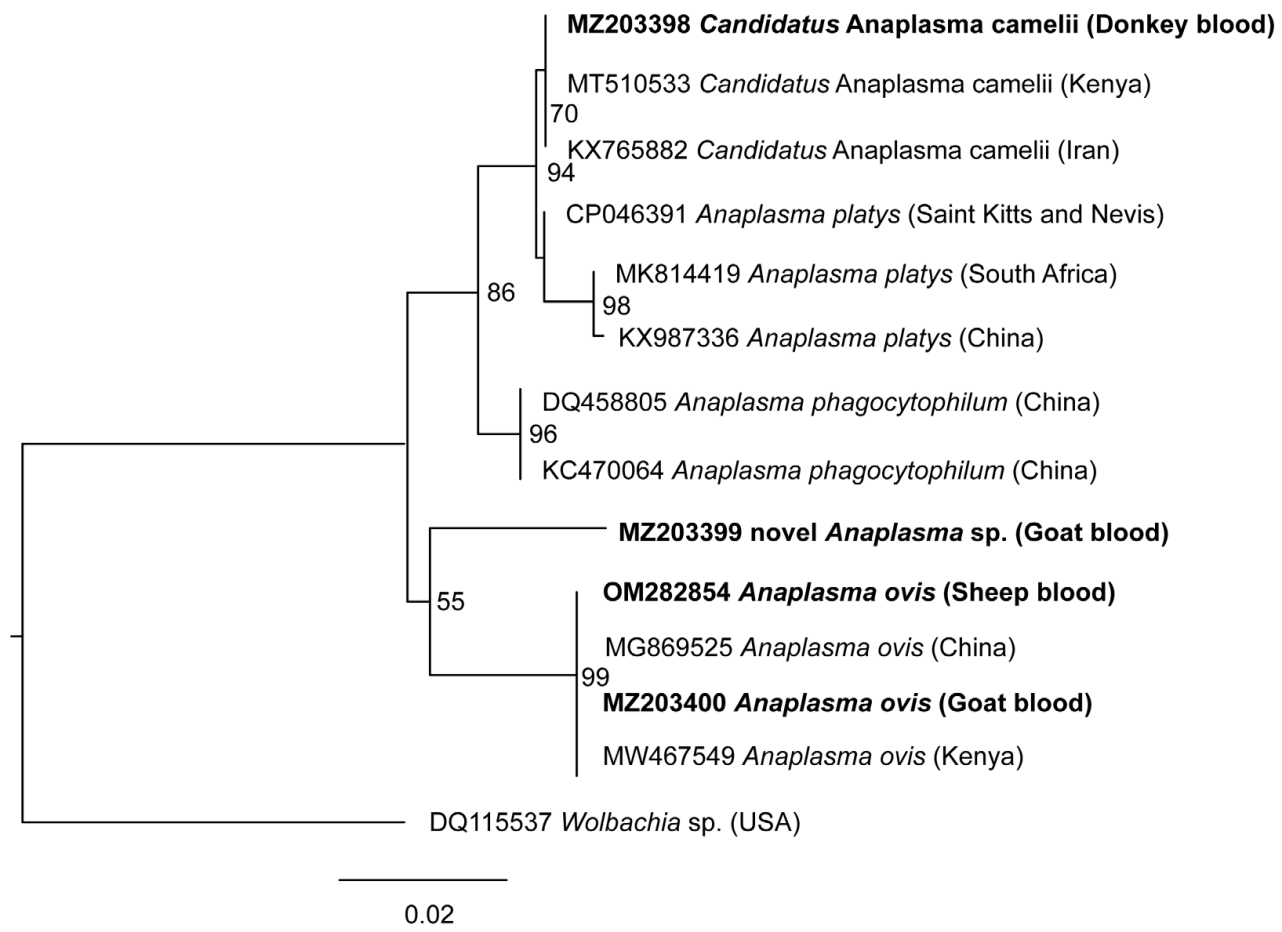
Maximum likelihood phylogenetic tree of 16S rRNA gene of *Anaplasma* spp. The tree shows the close relation as well as genetic divergence of sequences from this study and that queried from GenBank. Bootstrap values at the major nodes are of percentage agreement among 1000 bootstrap replicates. GenBank accession numbers, species identification and country of origin are indicated for each sequence. Sequences from this study are indicated in bold, and the associated sample indicated at the end. *Wolbachia* endosymbiont (GenBank accession DQ115537) 16S rRNA was used as the outgroup.

The phylogenetic tree comparing sequences of 16S rRNA gene fragments of *Anaplasma* (900–1000 bp) from this study to other sequences of the same gene available in GenBank is presented in ( Figure 4). Phylogenetic relationships and molecular evolution were inferred using the maximum likelihood method. Tree Topologies were estimated using nearest neighbor interchange improvements over 1,000 bootstrap replicates. The tree was drawn to scale representing a 2% evolutionary change in nucleotides per site.

## Discussion

There is little information about infectious pathogens, particularly zoonotic, circulating in livestock of northern Kenya, due to lack of proper disease surveillance. In the area of study in Laisamis, northern Kenya, we aimed to determine the occurrence and prevalence of selected hemopathogens in livestock (goats, sheep and donkeys) and their predominant ectoparasitic keds (collected from goats, sheep, donkeys and dogs).

Our findings revealed *T. vivax* as the predominant trypanosome species in livestock and keds, outside the tsetse belts, with an infection rate of 6.7% (26/389) and 22.6% (53/235), respectively. This agrees with a previous report that showed *T. vivax* as the major case of trypanosome infection outside tsetse-infested areas in western Kenya ( Thumbi *et al.*, 2010). This species is known to be pathogenic to goats, sheep and equids ( Galiza *et al.*, 2011). Similarly, *T. vivax* infection in camels and camel keds was previously reported from the same study area ( Kidambasi *et al.*, 2020), suggesting *T. vivax* as the major cause of trypanosomiasis in Laisamis, northern Kenya. Trypanosomiasis disease caused by this pathogen is among the most important diseases limiting livestock productivity and agricultural development, for example in Ethiopia ( Bedada & Dagnachew, 2012).

Donkeys harbored *T. vivax*, *E. canis*, ‘ *Ca*. Anaplasma camelii’, and *T. equi*, with *E. canis* being the most prevalent pathogen 25% (9/36). *E. canis* is receiving increasing attention due to its high morbidity and mortality in animals ( Bunroddith *et al.*, 2018). Detection of the canine pathogen, *E. canis*, in donkeys is not surprising because the pastoralist farmers rear mixed livestock species together with other domestic animals including dogs; thus, the donkeys could have acquired this pathogen from infected dogs through insect or tick bites. Ehrlichiosis is an emerging disease of domestic animals mainly transmitted by ticks and has previously been reported to infect dogs, cattle, humans, and goats ( Zhang *et al.*, 2017).

Further, donkeys were found to be infected with the camel-associated bacteria, ‘ *Ca.* Anaplasma camelii’, previously reported in Kenyan camels ( Kidambasi *et al.*, 2020). The presence of this camel pathogen in donkeys could be attributed to co-herding of donkeys with camels, and the donkeys could have acquired the pathogen from infected camels. Keds are common ectoparasites infesting livestock in the study area and have been reported as mechanical vectors of this bacterial pathogen ( Bargul *et al.*, 2021). Further research is needed to determine the zoonotic potential as well as the pathogenic role of this pathogen in donkeys and other livestock species. *T. equi* (13.9%) was also detected in donkeys. Pathogens associated with this genus are among the causative agents of equine piroplasmosis and have previously been shown to infect donkeys in some parts of Kenya including Mwingi ( Oduori *et al.*, 2015).

Goats harbored *T*. *vivax*, *E. canis*, *T. ovis*, *A*. *ovis* and a novel *Anaplasma* species. Sheep were also found to harbor *E. canis*, *T. ovis,* and *A. ovis. T. ovis* was more prevalent in sheep (38.9%) than in goats (0.8%). This finding is consistent with a study done on livestock in Palestine ( Azmi *et al.*, 2019). Ovine theileriosis caused by *T. ovis* is among the most important infectious diseases affecting small ruminants, leading to significant economic losses to farmers ( Al-Hosary *et al.*, 2021). *E. canis* and *T. vivax* were detected in most of the samples analyzed, including keds, suggesting these two are the common pathogens circulating in livestock herds in the study area. Further research will be needed to understand the vectors of these pathogens and whether keds are competent vectors of the pathogens.

Additionally, a high prevalence of *A. ovis* was detected in goats (84.5%) and sheep (93.5%), which is consistent with findings in a study done in Tunisia, North Africa, in goats and sheep by PCR ( Said *et al.*, 2015). This high prevalence of *A. ovis* could also be attributed to ticks that were present in most of the domestic animals. *A. ovis* is distributed worldwide and considered a major cause of small ruminant anaplasmosis in tropical and subtropical regions of the world, with general clinical effects ranging from fever, fatigue, low milk production and abortion but with a low mortality rate ( Stuen & Longbottom, 2011). A previous study in Corsica, France, reported the presence of *A. ovis* in dairy goats after an extensive survey due to health and production problems encountered in the goat flocks ( Cabezas-Cruz *et al.*, 2019b).

We discovered an unidentified *Anaplasma*-like species in 11.8% (29/245) of goats analysed. The *Anaplasma* sp. shared 96.77% sequence identity with *A. ovis* sequenced from goat blood in China (GenBank accession MG869525). The GC content of this novel *Anaplasma* sp. was 52.9% with clear observation of differences in the bases with the queried *Anaplasma* spp. from NCBI GenBank nr database ( Figure 3). The pathogenic role of this *Anaplasma*-like species in goats is not understood. However, it is related to *A. ovis*, which is known to be pathogenic in sheep, goats, and some wild ruminants ( Said *et al.*, 2015).

More hemopathogens were detected in dog keds than in other keds, and they include *T. vivax, T. evansi, T. simiae, E. canis, C. perfringens, B. schoenbuchensis,* and *B. abortus.* Due to the fact that most dogs had a free-roaming lifestyle in the study region, it is possible that dogs were at a higher risk of being infected with a wide range of pathogens. Therefore, keds collected from dogs acquired these pathogens from infected dogs during their bloodmeal feeding. We were unable to collect blood from dogs during sampling because we lacked proper protective gear against dog bites, and the dogs were not vaccinated from rabies; thus, collecting blood from dogs was considered too high-risk.

Among all the pathogens detected in keds obtained from dogs, *E. canis* (76%) and *B. schoenbuchensis* (76%) had the highest prevalence rates. The high prevalence rate of these pathogens could be attributed to the high competition of pathogens circulating in the same host population, considering the possible interactions between the pathogens and, host immune system, and host life cycle as well ( Poletto *et al.*, 2015). In previous studies, *B. schoenbuchensis* was also detected in deer ked by PCR test with a prevalence rate of more than 60% ( Szewczyk *et al.*, 2017). *B. schoenbuchensis* is one of the most important species that cause bartonellosis and has been reported to cause infections in humans, cattle and wild animals such as the cervids in Asia, North America and Europe ( Rolain *et al.*, 2003; Vayssier-Taussat *et al.*, 2016). Additionally, *Bartonella* infection often manifests as various cardiovascular, neurological and rheumatologic conditions, making it a public health concern since pastoralists, farmers and veterinarians who interact with domestic animals are at a high risk of infection ( Maggi *et al.*, 2012). There is little information on the presence of *E. canis* in keds, but this pathogen has been detected in *Rhipicephalus sanguineus* (the brown dog tick), the biological vector of the pathogen ( Cabezas-Cruz *et al.*, 2019a).

This study also reports the first occurrence of *C. perfringens* in dog keds. This pathogen is an important cause of enteric diseases in humans and domestic animals and is responsible for several forms of enterotoxaemia, which differs in clinical manifestation and severity according to the toxigenic type involved and specific toxins produced ( Singh *et al.*, 2018). It affects small ruminants worldwide, causing heavy mortality and significant economic impact ( Sumithra *et al.*, 2013). In previous reports, this pathogen has been shown to cause death in dogs due to hemorrhagic gastroenteritis of the gastrointestinal tract; thus further research is needed to better understand the role of this bacterium in enteric diseases of dogs ( Schlegel *et al.*, 2012).

The *Brucella* sp. (5.6%) detected in dog keds was closely related to *B. abortus* sequenced from cattle milk in India, showing a sequence identity of 98.68% (GenBank accession MK881176). Brucellosis in dogs is mainly associated with *B. canis* and not *B. abortus*, which mainly occur in cattle. However, cross-species transmission of *Brucella* spp. is possible and this is consistent with a study done in Argentina that detected *B. abortus* in farm dogs ( Mortola *et al.*, 2019). Additionally, *B. abortus* is a common source of human infection with a high zoonotic potential, and cause a disease called Bang’s disease in humans ( Kaden *et al.*, 2018). *Brucella* species have been shown to be of high public health and socio-economic importance in northern Kenya, and mainly transmitted to humans through ingestion of unpasteurized dairy products or raw/undercooked animal products ( Kairu-Wanyoike *et al.*, 2019).

Among trypanosomes detected in keds obtained from dogs, *T. vivax* (15.7%) was more prevalent than *T. evansi* (0.9%) and *T. simiae* (0.9%), which had low prevalence rates. The presence of these trypanosome species in dog keds suggests that the keds were infected during their bloodmeal acquisition from dogs that were initially infected with trypanosomes from ticks and possibly from other biting flies like *Stomoxys*. Additionally, the dogs had a free-roaming lifestyle and thus, it is also possible that they were infected when moving into neighboring tsetse-infested regions. Similarly, *T. vivax* (29.3%) was also more prevalent than *T. evansi* (0.86%) and *T. godfreyi* (0.86%) in keds obtained from goats. This low infection rate could be attributed to disease stability in the area, change in climate and seasonal outbreaks ( Gutierrez *et al.*, 2006). Both *T. vivax* and *T. evansi* have recently been detected in camel keds in northern Kenya ( Kidambasi *et al.*, 2020). Further, previous studies have shown *T. godfreyi* and *T. simiae* to infect a wide range of domestic animals including pigs, cattle, camels, dogs and goats with *T. simiae* being highly pathogenic to domestic pigs ( Hamill *et al.*, 2013; Simwango *et al.*, 2017). *T. simiae* and *T. godfreyi* are among the *Trypanosome* spp. that cause African animal trypanosomiasis with tsetse flies being their main vector ( Isaac *et al.*, 2016). This is the first report of occurrence of these two *Trypanosome* spp. in keds.

The molecular data from keds collected from donkeys showed detection of *T. vivax* (18.2%) and *E. canis* (63.6%) as the common pathogens. Detection of these pathogens in donkeys and goats, as well as in their associated ectoparasitic keds, shows the xenodiagnostic potential of using keds to indirectly screen for pathogens occurring in their associated hosts. Similarly, a recent report demonstrated the occurrence in keds of pathogens that were similarly present in their camel host from which they were collected, and further proposed the potential use of keds in xenodiagnosis ( Kidambasi *et al.*, 2020). However, detection of pathogens in keds does not incriminate them as vectors, but studies should be carried out to determine the vector competence of these keds. Keds are known to transmit mammalian trypanosomatidae of the genus *Megatrypanum* and are suspected to be vectors of *T. avium* and *T. corvi* in birds ( Svobodová *et al.*, 2015).

The impact of zoonotic pathogens is often underestimated due to limited surveillance and insufficient data of disease burden in most developing countries ( Munyua *et al.*, 2016). This study reveals that the domesticated animals, as well as keds collected from them, carried infectious pathogens of veterinary and public health concern. Notably, we sequenced multiple hemopathogens in dog keds, including zoonotic ones ( *B. abortus*, *B. schoenbuchensis*, and *C. perfringens*). Close association of humans with domestic animals infested by keds and other disease vectors increases chances of pathogen transmission. It is therefore crucial to conduct further studies to map out circulating livestock diseases in northern Kenya and establish the role of keds in disease transmission.

## Conclusions

We detected various selected infectious hemopathogens present in livestock and their associated ectoparasitic biting keds in northern Kenya, which calls for further surveillance studies to increase the understanding of the epidemiology of livestock diseases and the transmission of zoonotic ones by insect vectors such as keds that also occasionally feed on humans. This will guide the policy makers and livestock farmers in disease control.

## Data availability

### Underlying data

NCBI GenBank: Uncultured Anaplasma sp. clone An74 16S ribosomal RNA gene, partial sequence (16S rRNA of *Anaplasma ovis* in goat), accession number MZ203400: https://identifiers.org/ncbiprotein:MZ203400

NCBI GenBank: Uncultured Anaplasma sp. clone An8 16S ribosomal RNA gene, partial sequence (16S rRNA of novel *Anaplasma* sp. in goat), accession number MZ203399: https://identifiers.org/ncbiprotein:MZ203399

NCBI GenBank: Anaplasma ovis isolate 92B 16S ribosomal RNA gene, partial sequence (16S rRNA of *Anaplasma ovis* in sheep), accession number OM282854: https://identifiers.org/ncbiprotein:OM282854

NCBI GenBank: Candidatus Anaplasma camelii clone An4B 16S ribosomal RNA gene, partial sequence (16S rRNA of *Candidatus* Anaplasma camelii in donkey), accession number MZ203398: https://identifiers.org/ncbiprotein:MZ203398

NCBI GenBank: Uncultured Bartonella sp. clone EJ72 16S ribosomal RNA gene, partial sequence (16S rRNA of *Bartonella schoenbuchensis* in dog keds), accession number MZ203403: https://identifiers.org/ncbiprotein:MZ203403

NCBI GenBank: Uncultured Clostridium sp. clone Dg48 16S ribosomal RNA gene, partial sequence (16S rRNA of *Clostridium perfringens* in dog keds), accession number MZ203401: https://identifiers.org/ncbiprotein:MZ203401

NCBI GenBank: Trypanosoma simiae voucher T52 small subunit ribosomal RNA gene and internal transcribed spacer 1, partial sequence ( *T. simiae* ITS1 in dog keds), accession number MZ221829: https://identifiers.org/ncbiprotein:MZ221829

NCBI GenBank: Trypanosoma evansi voucher T64 small subunit ribosomal RNA gene and internal transcribed spacer 1, partial sequence ( *T. evansi* ITS1 in dog keds), accession number MZ221830: https://identifiers.org/ncbiprotein:MZ221830

NCBI GenBank: Ehrlichia canis isolate ES72 16S ribosomal RNA gene, partial sequence (Short 16S rRNA of *E. canis* in dog keds), accession number OM282855: https://identifiers.org/ncbiprotein:OM282855

NCBI GenBank: Theileria ovis isolate 26A small subunit ribosomal RNA gene, partial sequence (18S rRNA of *Theileria ovis* in sheep), accession number OM282856: https://identifiers.org/ncbiprotein:OM282856

NCBI GenBank: Theileria equi isolate 15D small subunit ribosomal RNA gene, partial sequence (18S rRNA of *Theileria equi* in donkey), accession number OM282857: https://identifiers.org/ncbiprotein:OM282857

Figshare: Detection of hemopathogens in goat, sheep, donkey, and their associated hematophagous biting keds, and dog keds, https://doi.org/10.6084/m9.figshare.18586028.v1 ( Mwaki *et al.*, 2022)

This project contains the following underlying data:

- Raw HRM Rotor Gene, Quant Studio and micPCR HRM data files for detection of pathogens in goats, sheep, donkeys and their associated biting keds, and dog keds. The HRM data files for *Anaplasma* spp. and *Ehrlichia* sp. can be accessed using Rotor Gene Q software, Quant Studio ^TM^ Design and Analysis software, and micPCR software, while data files for the other pathogens can be accessed using Quant Studio ^TM^ Design and Analysis software.

Data are available under the terms of the Creative Commons Zero "No rights reserved" data waiver (CC0 1.0 Public domain dedication).

## References

[ref-1] Al-Hosary AA ElSify A Salama AA : Phylogenetic study of *Theileria ovis* and *Theileria lestoquardi* in sheep from Egypt: Molecular evidence and genetic characterization. *Vet World.* 2021;14(3):634–639. 10.14202/vetworld.2021.634-639 33935408 PMC8076446

[ref-2] Azmi K Al-Jawabreh A Abdeen Z : Molecular Detection of *Theileria ovis* and *Theleiria equi* in Livestock from Palestine. *Sci Rep.* 2019;9(1):11557. 10.1038/s41598-019-47965-0 31399617 PMC6688999

[ref-3] Baldacchino F Muenworn V Desquesnes M : Transmission of pathogens by *Stomoxys* flies (Diptera, Muscidae): A review. *Parasite.* 2013;20:26. 10.1051/parasite/2013026 23985165 PMC3756335

[ref-4] Bargul JL Kidambasi KO Getahun MN : Transmission of ‘ *Candidatus* Anaplasma camelii’ to mice and rabbits by camel-specific keds, *hippobosca camelina*. *PLoS Negl Trop Dis.* 2021;15(8):e0009671. 10.1371/journal.pntd.0009671 34398891 PMC8389426

[ref-5] Bedada H Dagnachew S : Study on the prevalence of donkey trypanosomosis in Awi zone northwest Ethiopia. *Ethiop Vet J.* 2012;16(2). 10.4314/evj.v16i2.6

[ref-6] Bunroddith K Viseshakul N Chansiri K : QCM-based rapid detection of PCR amplification products of *Ehrlichia canis*. *Anal Chim Acta.* 2018;1001:106–111. 10.1016/j.aca.2017.10.037 29291792

[ref-7] Cabezas-Cruz A Allain E Ahmad AS : Low genetic diversity of *Ehrlichia canis* associated with high co-infection rates in *Rhipicephalus sanguineus (s.l.)*. *Parasit Vectors.* 2019a;12(1):12. 10.1186/s13071-018-3194-9 30616670 PMC6322249

[ref-8] Cabezas-Cruz A Gallois M Fontugne M : Epidemiology and genetic diversity of *Anaplasma ovis* in goats in Corsica, France 06 Biological Sciences 0604 Genetics. *Parasit Vectors.* 2019b;12(1). 10.1186/s13071-018-3269-7 PMC6318933 30606253

[ref-9] Ereqat S Nasereddin A Vayssier-Taussat M : Molecular evidence of *Bartonella* species in ixodid ticks and domestic animals in palestine. *Front Microbiol.* 2016;7:1217. 10.3389/fmicb.2016.01217 27540374 PMC4972812

[ref-10] Galiza GJN Garcia HA Assis ACO : High mortality and lesions of the central nervous system in Trypanosomosis by *Trypanosoma vivax* in Brazilian hair sheep. *Vet Parasitol.* 2011;182(2–4):359–63. 10.1016/j.vetpar.2011.05.016 21664764

[ref-11] Getahun MN Villinger J Bargul JL : Molecular characterization of pathogenic African trypanosomes in biting flies and camels in surra-endemic areas outside the tsetse fly belt in Kenya. *bioRxiv.* 2020. 10.1101/2020.06.18.156869

[ref-12] Gubbels JM De Vos AP Van Der Weide M : Simultaneous detection of bovine *Theileria* and *Babesia* species by reverse line blot hybridization. *J Clin Microbiol.* 1999;37(6):1782–9. 10.1128/JCM.37.6.1782-1789.1999 10325324 PMC84950

[ref-13] Guindon S Dufayard JF Lefort V : New algorithms and methods to estimate maximum-likelihood phylogenies: Assessing the performance of PhyML 3.0. *Syst Biol.* 2010;59(3):307–21. 10.1093/sysbio/syq010 20525638

[ref-14] Gutierrez C Corbera JA Morales M : Trypanosomosis in goats: Current status. *Ann N Y Acad Sci.* 2006;1081:300–10. 10.1196/annals.1373.040 17135529

[ref-15] Hamill LC Kaare MT Welburn SC : Domestic pigs as potential reservoirs of human and animal trypanosomiasis in Northern Tanzania. *Parasit Vectors.* 2013;6(1):322. 10.1186/1756-3305-6-322 24499540 PMC3843548

[ref-16] Isaac C Ciosi M Hamilton A : Molecular identification of different trypanosome species and subspecies in tsetse flies of northern Nigeria. *Parasit Vectors.* 2016;9(1):301. 10.1186/s13071-016-1585-3 27216812 PMC4877947

[ref-17] Jones KE Patel NG Levy MA : Global trends in emerging infectious diseases. *Nature.* 2008;451(7181):990–3. 10.1038/nature06536 18288193 PMC5960580

[ref-18] Kaden R Ferrari S Jinnerot T : *Brucella abortus*: Determination of survival times and evaluation of methods for detection in several matrices. *BMC Infect Dis.* 2018;18(1):259. 10.1186/s12879-018-3134-5 29871600 PMC5989407

[ref-19] Kairu-Wanyoike S Nyamwaya, D Wainaina M : Positive association between *Brucella* spp. Seroprevalences in livestock and humans from a cross-sectional study in Garissa and Tana River Counties, Kenya. *PLoS Negl Trop Dis.* 2019;13(10):e0007506. 10.1371/journal.pntd.0007506 31622339 PMC6818805

[ref-20] Kearse M Moir R Wilson A : Geneious Basic: An integrated and extendable desktop software platform for the organization and analysis of sequence data. *Bioinformatics.* 2012;28(12):1647–9. 10.1093/bioinformatics/bts199 22543367 PMC3371832

[ref-21] Kiara H Jennings A Bronsvoort BMDC : A longitudinal assessment of the serological response to *Theileria parva* and other tick-borne parasites from birth to one year in a cohort of indigenous calves in western Kenya. *Parasitology.* 2014;141(10):1289–98. 10.1017/S003118201400050X 24838078 PMC4113304

[ref-22] Kidambasi KO Masiga DK Villinger J : Detection of blood pathogens in camels and their associated ectoparasitic camel biting keds, *Hippobosca camelina:* the potential application of keds in xenodiagnosis of camel haemopathogens [version 2; peer review: 2 approved]. *AAS Open Res.* 2020;2:164. 10.12688/aasopenres.13021.2 32510036 PMC7243205

[ref-23] Maggi RG Mozayeni BR Pultorak EL : *Bartonella* spp. Bacteremia and rheumatic symptoms in patients from Lyme disease-endemic region. *Emerg Infect Dis.* 2012;18(5):783–91. 10.3201/eid1805.111366 22516098 PMC3358077

[ref-24] Malabo Montpellier Panel: Meat, Milk and More : Policy innovations to shepherd inclusive and sustainable livestock systems in Africa. Malabo Montepellier Panel.2020;94. Reference Source

[ref-25] Marsabit CIDP: Marsabit County Integrated Development Plan 2018-2022. Second County Integrated Development Plan 2018-2022.2018. Reference Source

[ref-26] Mburu S Otterbach S Sousa-Poza A : Income and Asset Poverty among Pastoralists in Northern Kenya. *J Dev Stud.* 2017;53(6):971–986. 10.1080/00220388.2016.1219346

[ref-27] Mortola E Miceli GS Meyer LP : *Brucella abortus* in Dog Population: An Underestimated Zoonotic Disease. *Biomed J Sci & Tech Res.* 2019;15(2). 10.26717/BJSTR.2019.15.002681

[ref-28] Munyua P Bitek A Osoro E : Prioritization of zoonotic diseases in Kenya, 2015. *PLoS One.* 2016;11(8):e0161576. 10.1371/journal.pone.0161576 27557120 PMC4996421

[ref-29] Mwaki D Kidambasi K Kinyua J : Detection of hemopathogens in goats, sheep, donkeys, and their associated hematophagous biting keds, and dog keds (genus Hippobosca). *figshare.* Dataset. 2022. 10.6084/m9.figshare.18586028.v1 PMC10314185 37396343

[ref-30] Mwamuye MM Kariuki E Omondi D : Novel Rickettsia and emergent tick-borne pathogens: A molecular survey of ticks and tick-borne pathogens in Shimba Hills National Reserve, Kenya. *Ticks Tick Borne Dis.* 2017;8(2):208–218. 10.1016/j.ttbdis.2016.09.002 28011185

[ref-31] Narladkar BW : Projected economic losses due to vector and vector-borne parasitic diseases in livestock of india and its significance in implementing the concept of integrated practices for vector management. *Vet World.* 2018;11(2):151–160. 10.14202/vetworld.2018.151-160 29657396 PMC5891867

[ref-32] Njiru ZK Constantine CC Guya S : The use of ITS1 rDNA PCR in detecting pathogenic African trypanosomes. *Parasitol Res.* 2005;95(3):186–192. 10.1007/s00436-004-1267-5 15619129

[ref-33] Nyariki DM Amwata DA : The value of pastoralism in Kenya: Application of total economic value approach. *Pastoralism.* 2019;9(1). 10.1186/s13570-019-0144-x

[ref-34] Oduori DO Onyango SC Kimari JN : A field survey for the seroprevalence of *Theileria equi* and *Babesia caballi* in donkeys from Nuu Division, Kenya. *Ticks Tick Borne Dis.* 2015;6(5):683–8. 10.1016/j.ttbdis.2015.05.015 26072000

[ref-35] Otte J Pica-Ciamarra U Morzaria S : A comparative overview of the livestock-environment interactions in Asia and Sub-Saharan Africa. *Front Vet Sci.* 2019;6:37. 10.3389/fvets.2019.00037 30854375 PMC6395385

[ref-36] Oyieke FA Reid G : The Mechanical transmission of *Trypanosoma evansi* by Haematobia minuta ( Diptera : Muscidae ) and the survival of Trypanosomes in fly mouthparts parts and gut (A Preliminary Record). *Folia Veterinaria.* 2003;47(1).

[ref-37] Parola P Roux V Camicas JL : Detection of ehrlichiae in African ticks by polymerase chain reaction. *Trans R Soc Trop Med Hyg.* 2000;94(6). 10.1016/s0035-9203(00)90243-8 11198664

[ref-38] Perry B Grace D : The impacts of livestock diseases and their control on growth and development processes that are pro-poor.In *Philosophical Transactions of the Royal Society B: Biological Sciences.* 2009;364(1530). 10.1098/rstb.2009.0097 PMC2865091 19687035

[ref-39] Petersen FT Meier R Kutty SN : The phylogeny and evolution of host choice in the Hippoboscoidea (Diptera) as reconstructed using four molecular markers. *Mol Phylogenet Evol.* 2007:45(1):111–22. 10.1016/j.ympev.2007.04.023 17583536

[ref-40] Poletto C Meloni S Van Metre A : Characterising two-pathogen competition in spatially structured environments. *Sci Rep.* 2015;5:7895. 10.1038/srep07895 25600088 PMC4298724

[ref-41] Probert WS Schrader KN Khuong NY : Real-Time Multiplex PCR Assay for Detection of *Brucella* spp., *B. abortus,.* and *B. melitensis.* *J Clin Microbiol.* 2004;42(3):1290–3. 10.1128/JCM.42.3.1290-1293.2004 15004098 PMC356861

[ref-42] Rahola N Goodman SM Robert V : The Hippoboscidae (insecta: Diptera) from Madagascar, with new records from the “parc National de Midongy Befotaka.” *Parasite.* 2011;18(2):127–40. 10.1051/parasite/2011182127 21678788 PMC3671411

[ref-43] Rambaut A : FigTree. version 1.4.3. Institute of Evolutionary Biology, University of Edinburgh.2016.

[ref-44] Rolain JM Rousset E La Scola B : *Bartonella schoenbuchensis* isolated from the blood of a French cow. *Ann N Y Acad Sci.* 2003;990:236–8. 10.1111/j.1749-6632.2003.tb07370.x 12860633

[ref-45] Rosenberg R Lindsey NP Fischer M : Vital Signs: Trends in Reported Vectorborne Disease Cases — United States and Territories, 2004-2016. *MMWR Morb Mortal Wkly Rep.* 2018;67(17):496–501. 10.15585/mmwr.mm6717e1 29723166 PMC5933869

[ref-46] Said MB Belkahia H Alberti A : Molecular survey of *Anaplasma* species in small ruminants reveals the presence of novel strains closely related to *A. phagocytophilum* in Tunisia.In *Vector Borne Zoonotic Dis.* 2015;15(10):580–90. 10.1089/vbz.2015.1796 26394065 PMC4593892

[ref-47] Schlegel BJ Van Dreumel T Slavić D : *Clostridium perfringens* type a fatal acute hemorrhagic gastroenteritis in a dog. *Can Vet J.* 2012;53(5):555–7. 23115371 PMC3327598

[ref-48] Simwango M Ngonyoka A Nnko HJ : Molecular prevalence of trypanosome infections in cattle and tsetse flies in the Maasai Steppe, northern Tanzania. *Parasit Vectors.* 2017;10(1):507. 10.1186/s13071-017-2411-2 29061160 PMC5654092

[ref-49] Singh DD Pawaiya RS Gururaj K : Molecular detection of *Clostridium perfringens* toxinotypes, enteropathogenic *Escherichia coli,* rotavirus and coronavirus in diarrheic fecal samples of neonatal goat kids. *Veterinarski Arhiv.* 2018;88(1):1–20. 10.24099/VET.ARHIV.161027

[ref-50] Stuen S Longbottom D : Treatment and Control of Chlamydial and Rickettsial Infections in Sheep and Goats.In *Vet Clin North Am Food Anim Pract.* 2011;27(1):213–233. 10.1016/j.cvfa.2010.10.017 21215905

[ref-51] Sumithra TG Chaturvedi VK Siju SJ : Enterotoxaemia in goats—A review of current knowledge.In *Small Ruminant Research.* 2013;114(1):1–9. 10.1016/j.smallrumres.2013.05.013

[ref-52] Svobodová M Volf P Votýpka J : Trypanosomatids in ornithophilic bloodsucking Diptera. *Med Vet Entomol.* 2015;29(4):444–7. 10.1111/mve.12130 26211924

[ref-53] Szewczyk T Werszko J Steiner-Bogdaszewska Z : Molecular detection of *Bartonella* spp. in deer ked ( *Lipoptena cervi*) in Poland. *Parasit Vectors.* 2017;10(1):487. 10.1186/s13071-017-2413-0 29037227 PMC5644074

[ref-54] Thumbi SM Jung’A JO Mosi RO : Spatial distribution of African animal trypanosomiasis in suba and teso districts in Western Kenya. *BMC Res Notes.* 2010;3:6. 10.1186/1756-0500-3-6 20205857 PMC2826354

[ref-55] Tokarz R Kapoor V Samuel JE : Detection of tick-borne pathogens by masstag polymerase chain reaction. *Vector Borne Zoonotic Dis.* 2009;9(2):147–52. 10.1089/vbz.2008.0088 18800864 PMC2976645

[ref-56] Vayssier-Taussat M Moutailler S Féménia F : Identification of novel zoonotic activity of *Bartonella spp,* France. *Emerg Infect Dis.* 2016;22(3):457–62. 10.3201/eid2203.150269 26885624 PMC4766919

[ref-57] Wu J Zhang W Xie B : Detection and toxin typing of *Clostridium perfringens* in formalin-fixed, paraffin-embedded tissue samples by PCR. *J Clin Microbiol.* 2009;47(3):807–810. 10.1128/JCM.01324-08 19109478 PMC2650962

[ref-58] Zhang H Chang Z Mehmood K : First report of *Ehrlichia* infection in goats, China. *Microb Pathog.* 2017;110:275–278. 10.1016/j.micpath.2017.07.012 28705746

