# Supplementary material for: Molecular detection of novel Anaplasma sp . and zoonotic hemopathogens in livestock and their hematophagous biting keds (genus Hippobosca) from Laisamis, northern Kenya
Source: Open Res Afr. 2022 Jun 6;5:23. [Version 1] doi: 10.12688/openresafrica.13404.1 (PMC10314185; doi:10.12688/openresafrica.13404.1)
Supplement: Peer review history

# Peer review: Reviewer response for version 1

**DOI:** 10.21956/openresafrica.14552.r29852

**Published:** 2023-07-14

**Authors:** Agatha Kolo

**Affiliations:** 1 Molecular Microbiology and Immunology, The University of Texas at San Antonio, San Antonio, Texas, USA

This research paper was soundly written and provided information about the hemopathogens that were detected in sheep, goats, donkeys and their insect vectors and well as in keds collected from dogs using molecular tools in Northern Kenya. The study mostly cited the appropriate references, and I suggested a few that could be added to strengthen the points made in the discussion on the novel *Anaplasma* sp. detected in goats (please see the attached file linked here for additional comments).

 The only limitation I saw in the study was the fact that blood samples from dogs were not collected and screened in the study. Although the authors mentioned rabies risk as a factor that impeded sample collection from dogs, molecular screening on canine blood could have painted a clearer picture on the role of dogs as reservoirs of zoonotic pathogens and the public health risk posed to humans. Adequate planning with the use of dog catchers and appropriate PPE for sample collection should be put into consideration for future studies. In general, this research paper provided valuable information on the pathogens circulating in sheep, goats, donkeys and their keds and in dog keds in Laisamis, Northern Kenya.

Is the work clearly and accurately presented and does it cite the current literature?

Yes

If applicable, is the statistical analysis and its interpretation appropriate?

Not applicable

Are all the source data underlying the results available to ensure full reproducibility?

Yes

Is the study design appropriate and is the work technically sound?

Partly

Are the conclusions drawn adequately supported by the results?

Yes

Are sufficient details of methods and analysis provided to allow replication by others?

Yes

Reviewer Expertise:

Molecular parasitology, Genetic variation of zoonotic tick- borne diseases, Tick microbiome, Zoonotic vector-borne diseases, Phylogenetic analysis, Physiologic functions of tick bacterial endosymbionts and microbiome analysis.

I confirm that I have read this submission and believe that I have an appropriate level of expertise to confirm that it is of an acceptable scientific standard.

# Peer review: Reviewer response for version 1

**DOI:** 10.21956/openresafrica.14552.r29871

**Published:** 2023-06-30

**Authors:** Yasser F Elnaker

**Affiliations:** 1 Faculty of Veterinary Medicine, New Valley University, Kharga, New Valley Governorate, Egypt

In this article the authors noted that livestock and their associated ectoparasitic biting keds carry a number of infectious hemopathogens, including the zoonotic *B. abortus*. Dog keds harbored the most pathogens, suggesting dogs, which closely interact with livestock and humans, as key reservoirs of diseases in Laisamis. These findings can guide policy makers in disease control. The article is well-designed and gives good results about hem-pathogen in different animal app., in Kenya which may need further studies about its incidence and risk factors associated with each pathogen.

 Molecular biology is used easily in diagnosis and classification of pathogen. So, the article is good.

Is the work clearly and accurately presented and does it cite the current literature?

Yes

If applicable, is the statistical analysis and its interpretation appropriate?

Yes

Are all the source data underlying the results available to ensure full reproducibility?

Yes

Is the study design appropriate and is the work technically sound?

Yes

Are the conclusions drawn adequately supported by the results?

Yes

Are sufficient details of methods and analysis provided to allow replication by others?

Yes

Reviewer Expertise:

epidemiology & infectious diseases

I confirm that I have read this submission and believe that I have an appropriate level of expertise to confirm that it is of an acceptable scientific standard.

# Peer review: Reviewer response for version 1

**DOI:** 10.21956/openresafrica.14552.r29801

**Published:** 2023-06-27

**Authors:** Elisa Mazzotta

**Affiliations:** 1 Istituto Zooprofilattico Sperimentale delle Venezie (IZSVe), Legnaro, Italy

**Overall comments:**

 The work is well structured and provides information on the serological and molecular prevalence of various infectious and zoonotic pathogens in livestock reared in Laisamis, northern Kenya. The problem is well contextualised through appropriate bibliographical references. A direct detection of the investigated pathogens was carried out both in livestock and insect vector. Methods are accurately described. The results highlight the need for action on diseases that involve transmission by animal vectors and that involve other domestic and wild animal species in their cycle. The discussion deals with the topics in a clear and concise manner, also reporting the limitations of the study (e.g. it was not possible to take blood samples from dogs for rabies risk), and the need to assess the competence of the vector in which the molecular tests found positive. Although the possibilities for intervention in such a particular environment are difficult, the work shows the need to implement surveillance to adequately guide the control of zoonotic diseases.

 I therefore believe that this article deserves to be indexed; I merely suggest completing the bibliography (see references).

Is the work clearly and accurately presented and does it cite the current literature?

Yes

If applicable, is the statistical analysis and its interpretation appropriate?

Not applicable

Are all the source data underlying the results available to ensure full reproducibility?

Yes

Is the study design appropriate and is the work technically sound?

Yes

Are the conclusions drawn adequately supported by the results?

Yes

Are sufficient details of methods and analysis provided to allow replication by others?

Yes

Reviewer Expertise:

Veterinary Science, Animal Health, Zoonosis

I confirm that I have read this submission and believe that I have an appropriate level of expertise to confirm that it is of an acceptable scientific standard.

## References

[1] : Keds, the enigmatic flies and their role as vectors of pathogens. *Acta Trop* .2020;209: 10.1016/j.actatropica.2020.105521 105521 10.1016/j.actatropica.2020.105521 32447028

[2] : First molecular evidence of Anaplasma ovis and Rickettsia spp. in keds (Diptera: Hippoboscidae) of sheep and wild ruminants. *Vector Borne Zoonotic Dis* .2011;11(10) : 10.1089/vbz.2011.0649 1319-21 10.1089/vbz.2011.0649 21923269

[3] : LOUSE FLIES, KEDS, AND RELATED FLIES ( Hippoboscoidea ).2002; 10.1016/B978-012510451-7/50019-0 349-362 10.1016/B978-012510451-7/50019-0

[4] : Bovine brucellosis - a comprehensive review. *Vet Q* .2021;41(1) : 10.1080/01652176.2020.1868616 61-88 10.1080/01652176.2020.1868616 33353489PMC7833053
